# You won’t believe what this guy is doing with the potato: The ObjAct stimulus-set depicting human actions on congruent and incongruent objects

**DOI:** 10.3758/s13428-021-01540-6

**Published:** 2021-02-25

**Authors:** Yarden Shir, Naphtali Abudarham, Liad Mudrik

**Affiliations:** 1 School of Psychological Sciences, Tel Aviv University, Ramat Aviv, POB 39040, 69978 Tel Aviv, Israel; 2 Sagol School of Neuroscience, Tel Aviv University, Tel Aviv, Israel

**Keywords:** Congruency, Scene, Object, Expectation violation, Context, Stimulus-set, DCNN, Image processing

## Abstract

Perception famously involves both bottom-up and top-down processes. The latter are influenced by our previous knowledge and expectations about the world. In recent years, many studies have focused on the role of expectations in perception in general, and in object processing in particular. Yet studying this question is not an easy feat, requiring—among other things—the creation and validation of appropriate stimuli. Here, we introduce the ObjAct stimulus-set of free-to-use, highly controlled real-life scenes, on which critical objects are pasted. All scenes depict human agents performing an action with an object that is either congruent or incongruent with the action. The focus on human actions yields highly constraining contexts, strengthening congruency effects. The stimuli were analyzed for low-level properties, using the SHINE toolbox to control for luminance and contrast, and using a deep convolutional neural network to mimic V1 processing and potentially discover other low-level factors that might differ between congruent and incongruent scenes. Two online validation studies (*N* = 500) were also conducted to assess the congruency manipulation and collect additional ratings of our images (e.g., arousal, likeability, visual complexity). We also provide full descriptions of the online sources from which all images were taken, as well as verbal descriptions of their content. Taken together, this extensive validation and characterization procedure makes the ObjAct stimulus-set highly informative and easy to use for future researchers in multiple fields, from object and scene processing, through top-down contextual effects, to the study of actions.

## Introduction

Despite what they might think, people do not see the world “as it is”: top-down processes, such as knowledge, memories, and feelings, affect what we perceive, as we continually evaluate our surroundings and act accordingly (e.g., Gilbert & Li, 2013; Gilbert & Sigman, 2007; Lupyan, 2015; but see Firestone & Scholl, 2016). Our experience provides us with knowledge, which makes us expect certain things based on their probability. For example, some of the knowledge we have on objects pertains to where they are likely to be found (Bar, 2004; Biederman, Mezzanotte, & Rabinowitz, 1982). Accordingly, when we see a scene, we expect particular objects to be in it, and when we see an object, we have expectations regarding the scene in which it appears. What happens when these expectations are violated?

Violations of expectations are defined as “incongruencies,” and when referring to objects in scenes, these expectations were suggested to be divided into five types (Biederman, 1981; see also Biederman et al., 1982): (a) we expect objects to obey the rules of gravity—meaning they should usually be supported by—or rest on—another object/the ground (i.e., we do not expect objects to float); (b) we expect the object to be in front of its setting, so that objects close to the observer will be fully presented and those farther behind will be hidden (i.e., we expect close objects to occlude distant ones); (c) we expect specific objects to appear in similar settings, meaning that objects that have appeared in a specific setting in the past are more likely to appear in it again, while objects that did not appear in a setting before will be unlikely to appear in it in the future (e.g., we expect to see knives and forks in the kitchen and not in the restroom); (d) we also expect the position of the object within the scene to be invariant to some degree, so it is more likely to appear in a specific location within the setting than in others (e.g., we expect the knives and forks to be placed on the table or in a drawer rather than on the floor); and (e) we expect objects to appear in their familiar size, which should additionally match the setting in which they are located (i.e., we expect them to preserve their relative size with respect to the other objects in the scene).

The most widely studied types of incongruencies have focused on objects’ probability of appearing in specific settings or within specific contexts. Such incongruencies have been studied in different ways. Some used isolated objects, which were either related or unrelated or positioned in the correct or incorrect order (Green & Hummel, 2006; Riddoch, Humphreys, Edwards, Baker, & Willson, 2003). Others focused on objects in scenes. There, some used line drawings (Biederman et al., 1982; De Graef, Christiaens, & D’Ydewalle, 1990; Henderson & Hollingworth, 1998; Hollingworth & Henderson, 2000, 2003), while others used real-life scenes that were either photographed (Coco, Nuthmann, & Dimigen, 2020; Proverbio & Riva, 2009; Underwood, Templeman, Lamming, & Foulsham, 2008; Underwood & Foulsham, 2006) or digitally edited (Davenport & Potter, 2004; Demiral, Malcolm, & Henderson, 2012; Draschkow, Heikel, Võ, Fiebach, & Sassenhagen, 2018; Underwood, Humphreys, & Cross, 2007), and there were also studies using videos (Sitnikova, Kuperberg, & Holcomb, 2003; Sitnikova, Holcomb, Kiyonaga, & Kuperberg, 2008). For the scenes, again, some manipulated the probability of the object being in the scene (e.g., Bonitz & Gordon, 2008; Zacharia, Ahuja, Kaur, Mehta, & Sharma, 2020), while others also manipulated its probability of being in a certain location in the scene (e.g., Võ & Wolfe, 2013), or the object’s obedience to the rules of gravity (e.g., Võ & Henderson, 2011).

But object-related incongruencies do not only stem from the scenes in which they appear. Rather, they can also be evoked by the ways objects are manipulated by human agents (or the motor plans—termed “affordances”—they evoke; Gibson, 1979). Accordingly, manipulating the likelihood of an object to be manipulated in a certain manner could affect both object processing and action comprehension. Such effects could rely on motor circuits involved in action comprehension (Calvo-Merino, Grèzes, Glaser, Passingham, & Haggard, 2006) or on semantic knowledge about the likelihood of objects being used in specific actions. For example, EEG studies have found a larger N400 component for actions performed on incongruent as opposed to congruent objects, using either videos (Sitnikova et al., 2008, Sitnikova et al., 2003; see also Amoruso, Finisguerra, & Urgesi, 2016 for a finding during the same time window using TMS) or photographs (Proverbio & Riva, 2009). This component, commonly held to represent integration processes or contextual expectation violations (Kutas & Federmeier, 2011), suggests that objects are harder to integrate, and perhaps even process, when inappropriately manipulated by human agents (see also Truman & Mudrik, 2018). Similarly, observing an action, even without the critical object being presented, facilitates the recognition of subsequently presented congruent versus incongruent objects (Helbig, Steinwender, Graf, & Kiefer, 2010). Taken together, these studies confirm that contextual effects are at play both for objects presented in scenes and for objects being manipulated by a human agent as part of an action.

Importantly, creating the stimuli to study questions of this sort is highly demanding, especially when using real-life images rather than line drawings; the researchers should control for the semantic probability of the objects appearing in the image, find the right objects and contexts (or take pictures of them), and combine them together. They should then validate the stimuli to make sure they are indeed judged by subjects according to the experimenters’ classifications. Finally, a further challenge is to equate—or at least control for—low-level properties of the stimuli. Thus, using existing stimulus sets could save researchers a substantial amount of time and effort. Currently, there are two stimulus sets that allow researchers to study the effect of context on scene perception (without human agents), and several sets pertaining to human action but without a congruency manipulation. For objects and scenes, the first stimulus set is the Berlin Object in Scene Database (BOis; Mohr et al., 2016), which presents cluttered scenes with target objects. However, it only manipulates objects’ probability of appearing in specific locations in the scene, and not their probability of appearing in that scene to begin with. Consequently it does not include stimuli where the target object does not belong in the scene (that is, incongruent objects), but only where the target object is placed in an unexpected location within the scene. The second set is the SCEGRAM Database for semantic and syntactic inconsistencies in scenes (Öhlschläger & Võ, 2017). There, congruent and incongruent objects are presented in scenes either at their correct locations or at incorrect locations (so both the probability and the location are manipulated). For objects in the context of actions, the stimulus sets typically include short videos as opposed to still images: the Moments in Time Dataset (Monfort et al., 2019) includes short videos presenting day-to-day human actions and events; the “something something” video database (Goyal et al., 2017) includes videos intended to be used to train neural networks, and contains short videos of actions which are meant to “make sense.” A stimulus set that does manipulate context was presented by Umla-Runge, Zimmer, Fu, and Wang (2012), where actions that are familiar in either Germany or China are presented. An additional stimulus set includes still images representing human actions (Delaitre, Laptev, & Sivic, 2010), yet again without any manipulation of object congruency. Recently, the stimuli used by Proverbio and Riva (2009), where semantic congruency was in fact manipulated, have been made available (the Event Task Stimulus Set; Riva, Dresang, Dickey, Warren, & Proverbio, 2020). However, these stimuli suffer from several shortcomings. First, the incongruencies are not restricted to the actions performed by the agent or to the interaction with the object (e.g., a man playing the violin underwater, a woman boxing while wearing a ballet costume, or a woman sitting in her pajamas between two men in suits). In fact, only 18 of the pictures present incongruencies pertaining to the use of an object within an action. Second, congruent and incongruent images are not matched, so researchers could not pair the stimuli (i.e., would be harder to control possible confounding variables). Finally, the validation procedure was limited to a group of ten judges, with no additional information collected on the images and no low-level analysis beyond mean luminance. Thus, there is currently no fully validated, highly detailed stimulus set with pairs of congruent and incongruent versions of human actions involving objects.

Here, we present such a stimulus set which directly targets action-based relations where human agents are performing an action involving either a congruent or an incongruent object. This set has several advantages: First, action-based context is highly constraining, inducing very strong expectations about the object; for scenes, there are typically many objects that are likely to appear, while for actions there is only one or very few options that are congruent with the scene (Mudrik, Lamy, & Deouell, 2010). Second, action-based relations might enjoy preferential processing compared with scene-based relations; some even claim that action-related, interacting objects are grouped perceptually (Green & Hummel, 2006; see also Humphreys & Riddoch, 2001).

Action-based relations were previously studied using both our own stimuli (13 papers overall; by our group: e.g., Biderman & Mudrik, 2018; Mudrik, Deouell, & Lamy, 2011; Mudrik & Koch, 2013; Truman & Mudrik, 2018; by other labs: Coco & Duran, 2016; Furtak et al., 2020; Mack, Clarke, Erol, & Bert, 2017; Moors, Boelens, van Overwalle, & Wagemans, 2016) and other stimuli (Dresang, Dickey, & Warren, 2019; Glanemann, Zwitserlood, Bölte, & Dobel, 2016; Proverbio & Riva, 2009; Rayner, Castelhano, & Yang, 2009). Yet, as we showed above, currently there is no organized, open-access stimulus set for action-based relations that has undergone a comprehensive validation and characterization procedure. Such a stimulus set would allow more refined questions to be tested (e.g., examining whether other factors, like emotional valence for example, are correlated with incongruency and might explain the results). In addition, the existing stimulus sets, including the original one we introduced in Mudrik et al. (2010), typically lack documentation of the source files from which the stimuli were created, which might lead to copyright issues. To meet all these requirements, we recreated our images while documenting their sources, including detailed information about the copyright status of the scenes and objects. In the current stimulus set, henceforth referred to as the ObjAct stimulus-set, all scenes and objects are defined as suitable for public use (at the time of stimuli creation). In addition, all images are classified and organized in an easy-to-use manner. Finally, we ran thorough validation experiments and analyzed the low-level features of all images; data for all stimuli are presented for future use by other researchers.

The ObjAct stimulus-set contains 120 pairs of images portraying a person performing an action with an object that has been replaced using Photoshop. In the congruent condition, the object was replaced with another object that fits the scene: either the same object taken from another source or a different object that is also likely to be found in the scene. In the incongruent condition, the original object was replaced with a different object that was unlikely to be found in the scene (e.g., a man taking a picture using a camera versus a porcupine, or a child washing a car using a sponge versus an ice cream sandwich). Thus, in both versions the critical object has been replaced by another object, either a congruent or an incongruent one.

Below we provide a full description of the presented stimulus set, as well as the results of several analyses and validation procedures we undertook to characterize the stimuli and collect different metrics on them. First, we conducted a low-level features analysis, examining possible low-level differences between congruent and incongruent images. This analysis was twofold. First, using the SHINE toolbox (Willenbockel et al., 2010), we looked for differences in luminance and contrast. Second, we tested for additional low-level differences using the outputs of the first convolution layer of the AlexNet deep convolutional neural network (DCNN) (Krizhevsky, Sutskever, & Hinton, 2012), which performs local operations of image analysis analogous to those of the primate primary visual area (V1) layer of the cortex. In two validation experiments, we presented two questions regarding the congruency of the image (i.e., the weirdness and likelihood of the images); we further presented questions to collect additional ratings of different aspects of the images (e.g., how disturbing or arousing is the image; see full details in Main validation questionnaire and Photoshop editing quality questionnaire sections). Based on these results, we further created three subsets of the stimulus set that can be useful for future researchers: the first (*N* = 100) includes pairs for which no low-level differences were found between the congruent and incongruent images; the second (*N* = 83) only includes images in which the critical object is man-made; and the third recombines the images to obtain new pairs of images (*N* = 22) where the critical object remains constant while the action changes to create congruent and incongruent images. Finally, we provide full documentation of the sources from which the stimuli were taken and of their copyright status, and all analysis codes and data which were used (https://osf.io/htnqd/)

## Methods and results

### Creating the stimulus set

The ObjAct stimulus-set and database can be downloaded from https://osf.io/htnqd/. It includes 120 pairs of congruent and incongruent images in which a person is performing an action on either an appropriate or an inappropriate object. All stimuli were created by digitally editing existing images rather than taking new photographs ourselves. This was done because our images involve an actual person performing an action with an object (as opposed to placing objects within stationary scenes). Thus, it would have been difficult to keep all features of the image identical between the congruent and incongruent versions when taking photographs: the people would have had to stand without moving and maintain the same facial expression after replacing the object they were holding (and make sure they still held it in exactly the same way). Therefore, we opted for editing the images. Notably, the original stimulus set included 121 pairs, but one was excluded after data collection (see Results section below).

#### Choosing images from the web

As the stimulus set is based on the one used in Mudrik et al. (2010), we first looked for new images to replace the old, undocumented ones, starting with the contexts (i.e., background). Using the Google Images search engine, we defined a filter of “Labeled for reuse with modifications,” to make sure all images were copyrighted for future use. Upon finding a suitable context image, we checked the privacy policy of the website in which the image was originally published to make sure the image was indeed free to use. Most images were downloaded for free from the publishing website, and some of them were purchased from stock images websites.

**Fig. 1:**
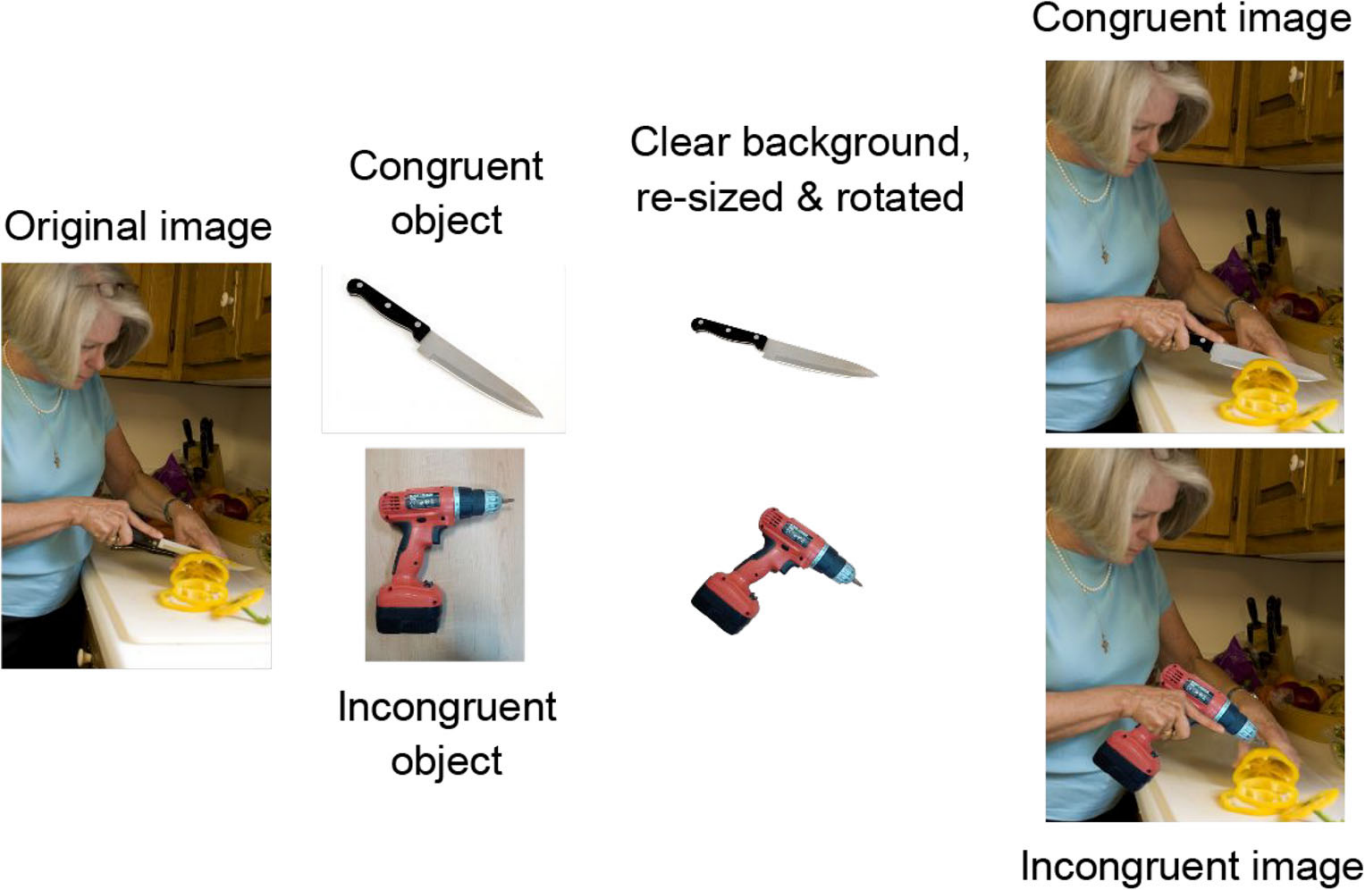
Example of creating stimuli. After finding a context image, we searched for congruent and incongruent objects. Those objects were then cut out of their backgrounds, re-sized, and rotated to match the context image

Then, for each context image, we searched for images of the congruent and incongruent objects, in an attempt to find the best fit in terms of size, lighting, sharpness, and orientation (e.g., if the object in the scene was oriented towards the person using it, we looked for other objects that would have a similar orientation). If a specific object was needed for several scenes, different exemplars of that object were chosen, so that the same image would only appear once in the full stimulus set (e.g., bottles appear in five scenes, and accordingly five different images of bottles were chosen). Importantly, we replaced the image of the congruent objects as well as the image of the incongruent objects, to minimize the difference between the two conditions as much as possible: we aimed for similar image-editing work in both conditions so as to avoid a confound between the conditions (see Fig. 1). Incongruent objects’ identity was chosen based on two main characteristics: first, the incongruent object needed to be of similar size as the congruent object to fit the scene, as we wanted to make sure the incongruency did not stem from a violation of expected size; second, the way in which the incongruent object was grasped had to be similar to that of the congruent object, again to make sure that the incongruency did not rest on the way the object was being held, but from its identity. Notably, after collecting all data, we discovered that images of two objects we used in the stimulus set were removed from their websites, which might evoke copyright issues; we thus recreated these two images while replacing the object, and provide the original images and the new ones in the stimulus set, while noting that the two new images were not included in the validation process. URL links to all images used (context images and objects) were documented and are listed in the Supplementary Table, which includes all details and specifications for the stimuli, and can be found on the website in which the stimuli are located. When using our stimuli in future studies, both this paper and the creators of the images you choose to display in your figures should be credited, as detailed in the Supplementary Table.

#### Editing the images

**Fig. 2:**
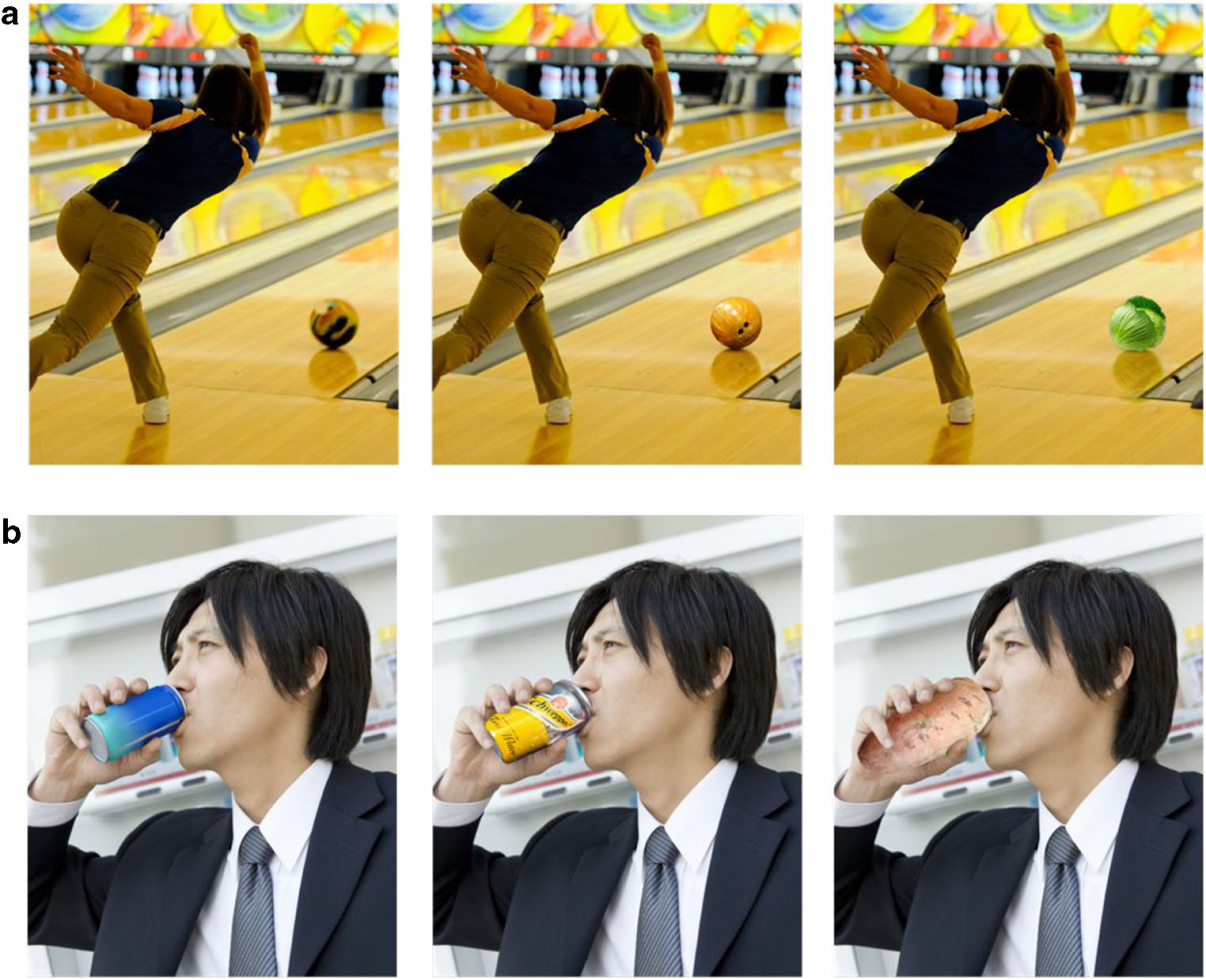
Examples of image digital editing. For each example, the original image (left), its congruent version (middle), and incongruent one (right) are portrayed. **a** Here, the reflection created by the object (ball/cabbage) was digitally modified to match the new objects. **b** Objects were not simply pasted onto the images, but further edited to avoid other violations of expectations beyond the probability of appearing in the image: here, the object (soda can/sweet potato) was cut so that the man’s fingers would appear in front of the object so that it would look like the man was holding it (image credit: **a** incongruent object: pngimg; **b** context image: Shutterstock)

All stimuli were edited by a professional designer using Adobe Photoshop CC 2017. All context images were first cut to a size of 440 × 550 screen pixels (note that these dimensions were based on the original stimulus set used by Mudrik et al., 2010); despite the relatively low resolution, the images can still be presented over relatively large visual angle fields and still seem clear). Then, images of congruent and incongruent objects were scaled and rotated to match the size and orientation of the original object as closely as possible, and luminance was modified if needed, based on the subjective impression of the designer. Each object was copied onto the context image as a new layer, so that both congruent and incongruent objects appeared as layers on the context image. This enabled precise and high-quality editing of objects and context images. If needed, the context image was edited as well (e.g., by removing parts of the original object that appeared in the scene and adding a matching background), in addition to editing the shades that should have been created by the objects, reflections, etc. (for examples, see Fig. 2a). Importantly, objects were cut out of their background and copied to match Biederman’s (1981) second type of expectations: they were edited to appear in front of their background but also behind other parts of the scene, if need be (e.g., if the context image depicted a man holding a potato, then the potato would appear in front of the man, but his fingers would appear in front of the potato, as they should be holding it; Fig. 2b). Using this method, we verified that the only difference manipulated between the congruent and incongruent conditions was the identity of the objects.

#### Supplementary Table

In addition to the images, we provide a summary table which includes information about the stimuli, along with the full statistical results. For a full description of the table and its column, see the supplementary information. In a nutshell, these include (a) descriptions of the context images and the objects; (b) image sources and credit requirements (notably, the information in the table is updated to June 2020, and it is the user’s responsibility to double-check that the sources are still valid); (c) results of the ranking questions in both questionnaires, as well as the multiple-choice question; (d) mean luminance; (e) mean contrast; (f) *x* and *y* coordinates of object location within the context image; (g) results of all statistical analyses, with false discovery rate (FDR) both corrected and uncorrected; and (h) raw data of DCNN analysis for each image and each filter. Notably, an additional classification of our stimuli relates to two types of incongruencies—an incompatibility between the object and the action itself (47 pairs), and an incompatibility between the object and the scene in which the action is taking place (73 pairs). To explain, in both cases the action itself does not make sense, so that even if the person were performing the action on a white background, it would be incongruent. In the latter type, however, the object is not only incongruent with the action, but also does not belong to the scene to begin with (as opposed to the Event Task Stimulus Set, where the object can be congruent with the action, but both the object and the action are incongruent with the scene, such as a man playing the trumpet in water; Riva et al., 2020). For example, in a bowling scene, it is unlikely that one would find a cabbage, and we accordingly classified this pair as scene-based; in another pair a man is taking a picture using a porcupine, which is unlikely even with no scene presented, and we accordingly classified this pair as agent-based. Thus, this information is included in the table to further allow researchers to make informed decisions about the specific types of stimuli they would like to use.

### Image analyses to control for low-level features

Possible low-level differences between the congruent and the incongruent images were evaluated using both the SHINE toolbox (Willenbockel et al., 2010) and the AlexNet deep convolutional neural network (DCNN; Krizhevsky et al., 2012). This evaluation was aimed at ensuring that effects found in studies using this stimulus set indeed stem from the congruency manipulation rather than from some systematic difference in low-level features between the stimuli. For example, if all congruent images are brighter than the incongruent images, then any effects evoked by these images might reflect brightness rather than congruency processing (for a related argument with respect to top-down effects on perception, see Firestone & Scholl, 2016). Notably, the original stimulus set introduced by Mudrik et al. (2010) was also validated, albeit to a much smaller extent; the stimuli were rated by 24 subjects with respect to their congruency level, without relating to other possible parameters. Also, the analysis of low-level properties conducted here is somewhat different from the original one, taking into account recent developments in image analysis. All analysis codes and data are publicly available and can be found on the Open Science Framework (OSF) website: https://osf.io/htnqd/.

#### SHINE

The SHINE toolbox (Willenbockel et al., 2010) was created to equate different visual features (e.g., luminance and contrast) between images. After trying to equate these factors, we noticed that the procedure introduced some distortion to the images. Thus, we opted not to modify the images using this toolbox in order to keep them in their natural form. However, future researchers are of course welcome to do so if they wish. Instead, we used SHINE’s imstats function to measure the mean luminance and contrast of each of the 240 images.

##### Luminance

**Fig. 3:**
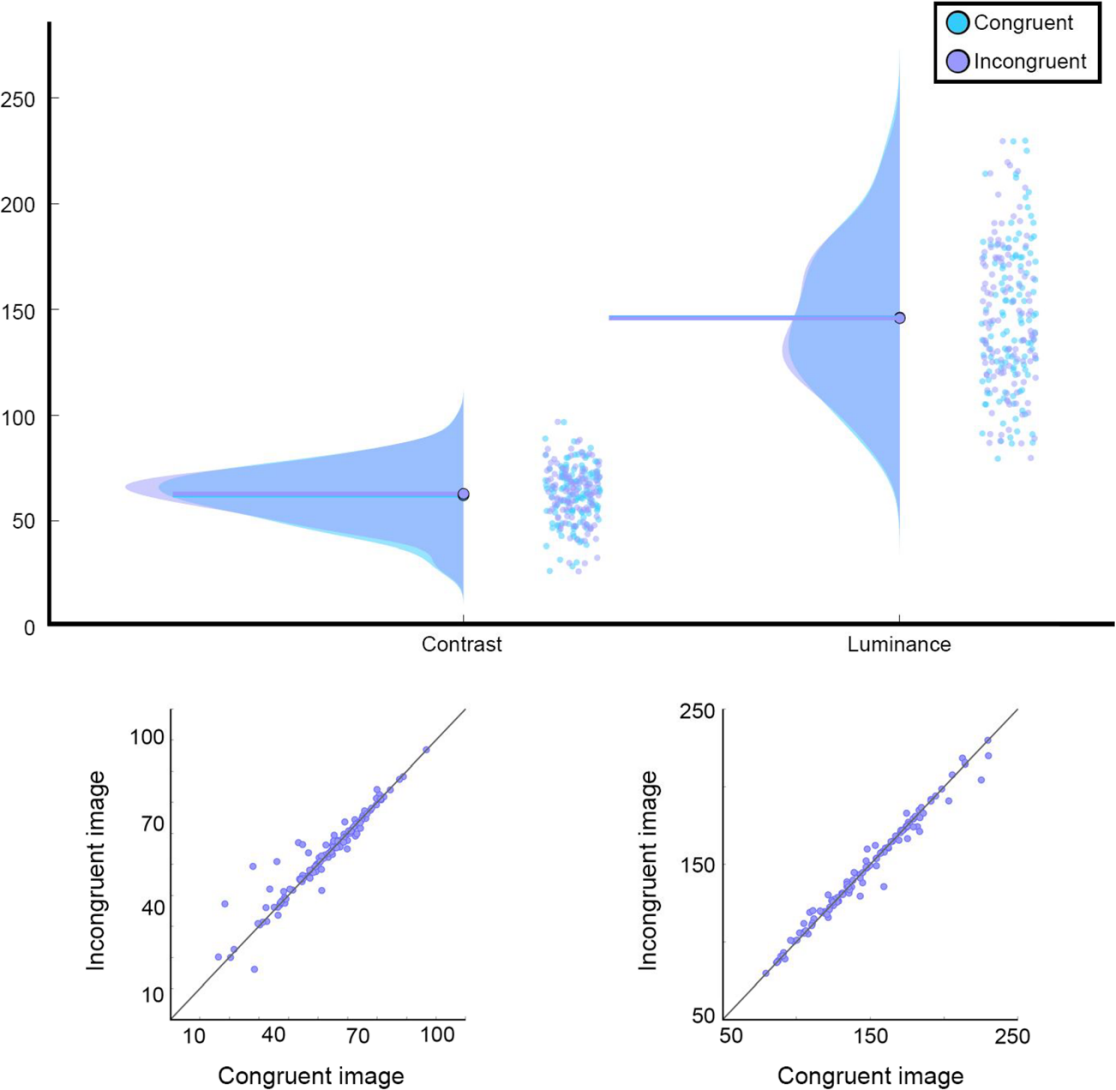
Top: distributions of mean contrast (left) and mean luminance (right) for congruent (cyan) and incongruent (purple) images. Thick lines represent the mean across all images. Individual dots represent individual images [plots were created using the raincloud plots (Allen, Poggiali, Whitaker, Marshall, & Kievit, 2019)]. Bottom: scatter plots of mean contrast (left) and mean luminance (right), where the mean value for congruent images is represented on the *x*-axis, and the mean value for incongruent images is represented on the *y*-axis. Accordingly, each individual dot represents one pair of images; the more the images are on the diagonal, the more similar the values for the congruent and incongruent images in that pair

The SHINE toolbox calculation of luminance is done by transforming each image to a matrix, in which the gray-level value for each pixel is saved. Then, the mean of the matrix is calculated as a measure of the image’s luminance. To compare the luminance between the image groups, we conducted a paired *t* test between the congruent and incongruent images, as well as a Bayesian paired *t* test, used with default settings and priors. The analysis showed no difference between congruent (*M* = 146.10, *SD* = 34.15) and incongruent images (*M* = 145.80, *SD* = 33.09; *t*(119) = 0.69, *p* = .595, BF_10_ = 0.128; see Fig. 3).

##### Contrast

To calculate contrast, the SHINE toolbox calculates the standard deviation of the matrix. The logic here is that a large variability in luminance between different pixels of the same image results in a high contrast, and vice versa. Here too, no difference was observed between congruent (*M* = 62.00, *SD* = 13.41) and incongruent (*M* = 62.72, *SD* = 12.84) images (*t*(119) = 1.96, *p* = .092, BF_10_ = 0.644; see again Fig. 3).

#### AlexNet DCNN

The analyses above focus on two specific low-level features, but there may be other low-level differences that are not revealed when taking such a top-down approach (where we decide in advance which features to test). To examine such differences from a bottom-up, more data-driven approach (with no a priori hypothesis about the types of differences that might be found), we fed the images into the AlexNet DCNN (Krizhevsky et al., 2012). DCNNs model the primate brain through the use of a hierarchical architecture, so that each layer in the hierarchy comprises multiple local convolution operations, operating on the whole field of view of the layer in a similar manner. Recent studies have found an analogy between these networks and both the primate V1 (Horikawa, Aoki, Tsukamoto, & Kamitani, 2019) and human visual cortex activity (Cichy, Khosla, Pantazis, Torralba, & Oliva, 2016; Eickenberg, Gramfort, Varoquaux, & Thirion, 2017; Khaligh-Razavi & Kriegeskorte, 2014). Therefore, their output in response to pairs of images can be used as an approximation of how similar the images are in terms of low-level information as perceived by humans. This accordingly allows us to look for systematic differences between the congruent and incongruent images in the different dimensions picked up by the network.

**Fig. 4:**
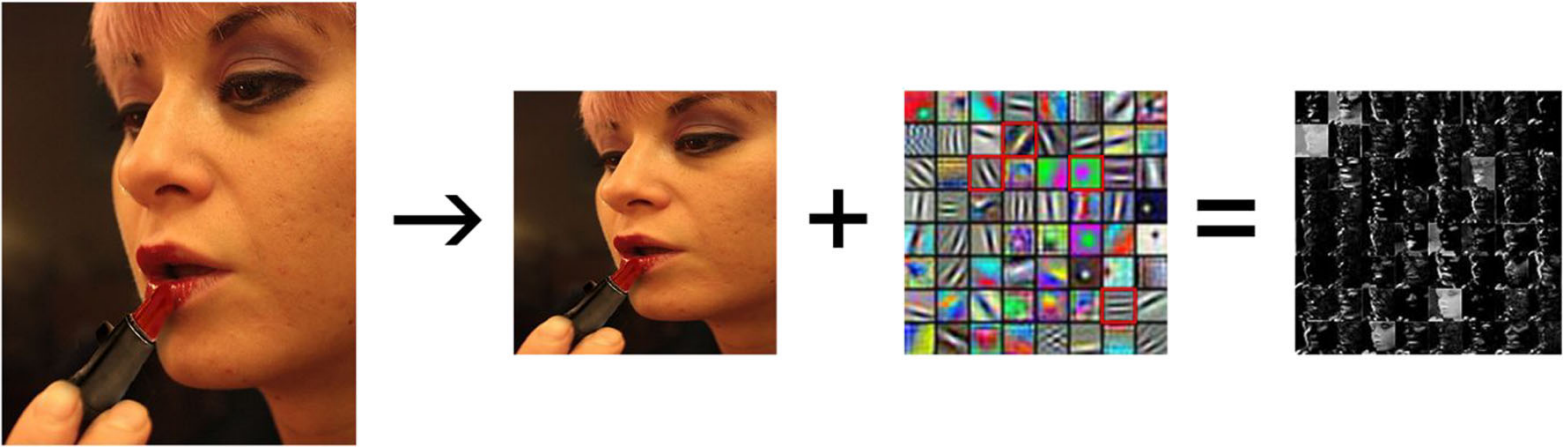
A visualization of the weights of the first layer of AlexNet. The image (left), resized to 224 × 224 pixels, combined with the 64 filters (middle), creates 64 matrices (visualized as 64 gray-level images, right). Each filter represents some low-level feature in the processed image. We took the mean of each matrix as a measure of the content of the corresponding feature, and used those values per image to calculate differences between congruent and incongruent images individually for each filter. Filters for which significant differences were found are marked by a red frame (image credit: Stephencdickson)

Figure 4 shows a visualization of the weights of the first layer of AlexNet, which is a DCNN that has been trained to classify objects, on the ImageNet data set (Deng et al., 2009). This first layer has 64 filters (convolutional kernels), 11 (width) by 11 (height) by 3 pixels (depth) in size. The weights of the filters are determined while the DCNN is trained to perform object classification, in an optimization process using the back-propagation algorithm (Krizhevsky et al., 2012). Visualizing the weights allows us to interpret the meaning of these convolution operations. For example, the first filter (top left square) has a red blob in its top left corner, indicating that this filter's response will be maximal for locations in the image with a similar pattern. Similarly, the last filter (bottom right square) has horizontal to diagonal black and white stripes, indicating that this filter's response will be maximal to Gabor-like patterns in similar frequency and orientation in the processed image. Therefore, the output of the first layer in AlexNet is the result of convoluting these 64 filters with the input image, thereby assessing the strength of this content in the image. For each filter, we can then calculate the mean values generated for all pixels of every image, and then compare these values between congruent and incongruent images.

To compare between the different images in our data set, we first resized the images to 224 × 224 pixel squares to accommodate the expected AlexNet file type. The model was then run on each image, and the output of the first AlexNet layer was used to calculate the activation of each filter on each image. This was defined as the mean value of the matrix resulting from the filter. Then, the outputs were compared using paired *t* tests as well as Bayesian *t* tests, separately for each filter. For 60 out of the 64 filters, no effect was found, while for the remaining four filters, we found a difference between congruent and incongruent images (see Supplementary Table for full details). Notably, this means that for the vast majority of the features, the congruent and incongruent images do not differ. However, three out of the four differences that were found relate to orientations that are more prominent in one group than the other, and the fourth difference pertains to the color distribution (purple vs. green on the left side of the image). Thus, for researchers who think that these differences might possibly confound their results, a subset of images for which no differences were found was created, as described below.

#### Low-level equated subset

Given the DCNN results above, we re-examined the set to see whether we could create a subset of the images for which no differences were found for any of the filters.

We used a jackknife-like method to reduce correlation biases driven by a single data point. In the original method, first introduced by Quenouille (1949), the analysis is conducted *N* times (where *N* equals the number of data points in the sample), so that in each run, one data point is removed from the sample to examine whether the effect depends on specific data points (in which case, no effect should be found when removing that data point). Similarly, here we looked for images which might drive the effect for the four DCNN filters. For each such filter, we removed the pairs with the largest differences between the congruent and incongruent versions. Then we reran both the SHINE and the DCNN analyses on the resulting subset, which included 100 pairs.

For this subset, no differences were found. This was true for all 64 DCNN filters (see details in the Supplementary Table), and for the luminance (*p* = .609) and contrast (*p* = .230) analyses. We additionally conducted all questionnaires analyses (see Image validation procedure section below) for these pairs only, and only corrected for multiple comparisons within this subset. Thus, the resulting subset, including 100 pairs, can be used by researchers as is, with all the metrics we have collected. The subset can be found in a separate folder in the stimulus-set website, alongside all results for the above analyses.

### Image validation procedure

In addition to the above low-level analyses, we ran a large-scale validation experiment to rate the congruency level of each image, thereby testing our manipulation and collecting more information about how subjects classify, interpret, and feel about the images. A separate questionnaire was conducted to evaluate the quality of the Photoshop editing. Here, too, all analysis codes and data can be found on the OSF website: https://osf.io/htnqd/.

#### Main validation questionnaire

The experiment was preregistered on the Open Science Framework, including the methods, sample size, and analysis plans. The preregistration can be found at: https://osf.io/htnqd/.

##### Methods

*Participants*. A total of 400 responses were collected to eight versions of the questionnaire (50 subjects per version, mean age = 39.5, 165 females and two who marked “other”). Participants were users of Amazon Mechanical Turk, who answer questionnaires online regularly, from either Canada or the USA. They all participated for payment ($5.5). All participants first signed an online consent form and passed a human verification test to make sure they were genuine participants. The study was approved by the ethics committee of Tel Aviv University. Sample size was determined to be 50 participants per image (as this is a validation study and we are not trying to confirm/disprove a theoretical claim or an empirical finding, we did not conduct any power analysis to determine the required sample size; instead, we chose a number which seemed large enough to yield reliable results), and we collected data until we reached that sample size. This number refers to valid subjects who met our exclusion criteria (see below).

*Subject exclusion*. Beyond the abovementioned 400 subjects, 35 participants were excluded because they did not complete the questionnaire, and eight additional participants were excluded because they failed in two or more of the catch questions. Two more participants were excluded because they wrote to us after finishing the questionnaire to tell us that they misunderstood one of the questions: when asked to say which object is most likely to appear in the image, those participants listed the object that was already presented in the image. Finally, one subject answered the same version of the questionnaire twice, so his second response was excluded.

*Apparatus and stimuli*. The questionnaire was created in Qualtrics. Participants answered the questions using their home computers. All 121 pairs of congruent and incongruent pairs were tested, presented in their original size (440 × 550 pixels). In each version of the questionnaire, 30–31 images were presented, half congruent and half incongruent. Two images from the same pair were never presented in the same questionnaire.

*Procedure*. The questionnaire had eight versions, each presenting 30–31 images (121 pairs of images or 242 images in total), with eight questions per image, and one additional “catch” question for three images in each version (“For quality control purposes, please select the number five here”*)*. The order of the images was randomly determined within each version. The questions were as follows: (a) “How weird is the image?” (b) “How likely is it to see such a scene in the real world?” (c) “How visually complicated is the image?” (d) “How hard is it to identify the object?” (here, the word “object” was replaced with the name of the specific object that appeared in the presented scene; for example, “How hard is it to identify the bottle?”). (e) “How disturbing is the image?” (f) “How much do you like the image?” (g) “How arousing do you find the image?” (h) “Which object is MOST likely to appear in the scene?” Questions (a)–(g) were answered using a seven-point Likert scale of 1 (*not at all*) to 7 (*very much*), and question (h) was a multiple-choice question, with the following options: the congruent object (e.g., bottle); the incongruent object (e.g., flashlight); another object that is semantically related to the congruent object (e.g., glass); another object that is semantically related to the incongruent object (e.g., candle); and “other” (with an option to fill in the answer). The four objects were randomly ordered, and the “other” option was always presented last. The purpose of this question was to assess the level of agreement across subjects about the most likely object to appear in the scene. The order of the questions was randomly determined for each image, to keep subjects alert and attentive and to avoid stereotypical responses.

##### Results

After collecting the data but before the analysis, one image pair was removed from the set (image pair number 50), since we realized that the designer had created it in a different way from the other scenes: the scene itself was a combination of two separate images rather than only one. All analyses below were accordingly performed without this image pair, over a total of 120 pairs. This scene is therefore no longer included in the stimulus set and is not described in the table.

The results of questions (a)–(g) were analyzed in two ways. First, confirmatory item-based analyses were conducted using independent *t* tests for each image pair individually: for each pair, all ratings by individual subjects (*N* = 50) were compared between the congruent and the incongruent versions. Results of the item-based analyses can be found in the Supplementary Table. Second, we decided to also perform exploratory between-group analyses (that were not preregistered) by comparing mean ratings for congruent versus incongruent images across subjects for each question. Similar to the low-level analyses, this was done using paired *t* tests as well as Bayesian statistics, relying on the Bayes factor measure with default settings and priors. That is, for each pair, the average ratings for the congruent and incongruent versions across subjects were calculated, and the resulting two vectors (*N* = 120) were compared. Here, too, all *p* values reported below are corrected for multiple comparisons using FDR (Benjamini & Hochberg, 1995).

**Fig. 5:**
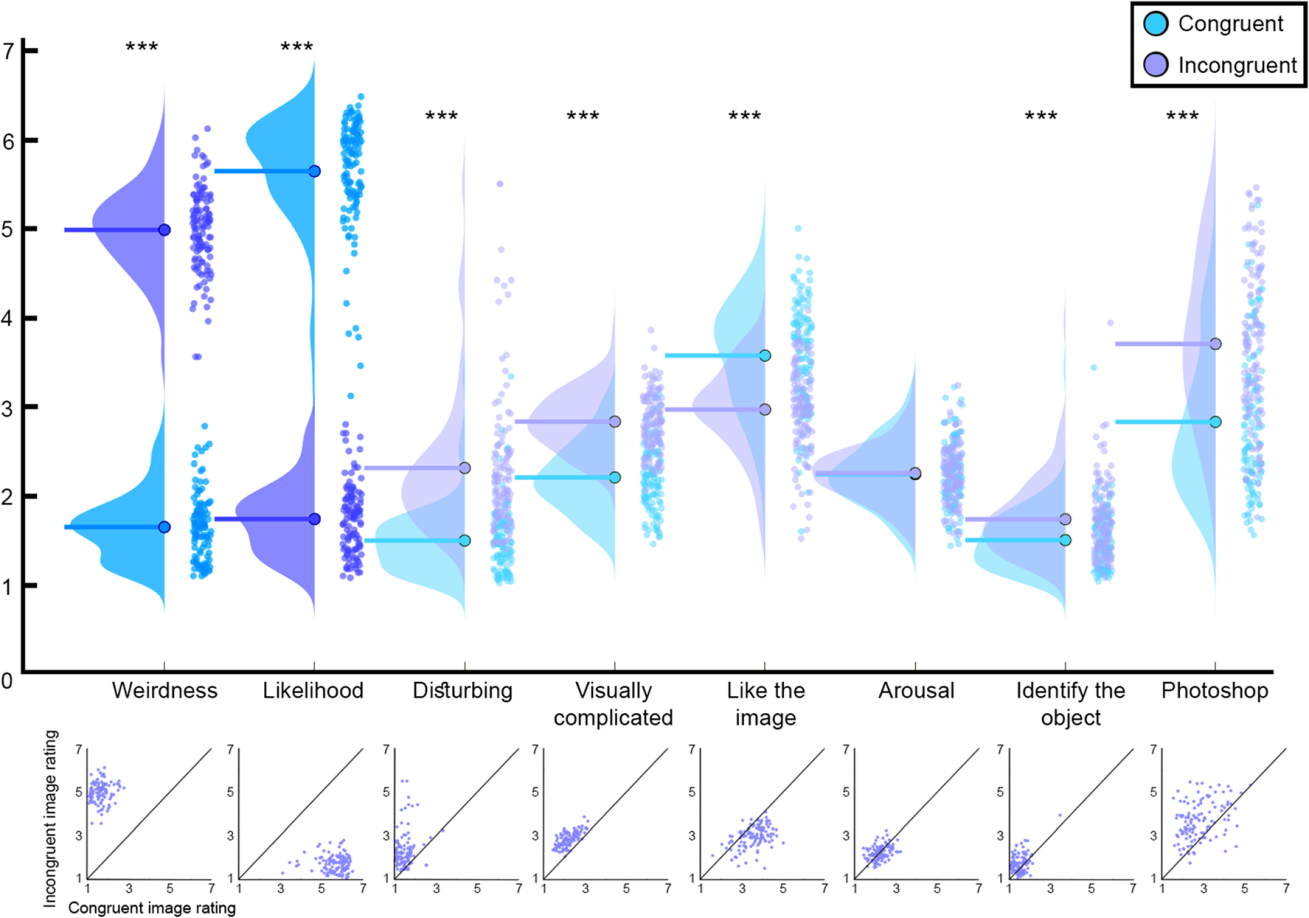
Results of the validation questionnaire for questions (a) to (g), and of the Photoshop editing quality questionnaire. The conventions are identical to those used in Fig. 3. Top: stronger colors represent the two main questions for measuring incongruency. Bottom: scatter plots of mean ratings for congruent (*x*-axis) and incongruent images (*y*-axis). For the full phrasing of the questions, see Methods section above (***p < .0001)

*Congruency-related questions*: Questions (a) (“How weird is the image?”) and (b) (“How likely is it to see such a scene in the real world?”) served as the key questions for validating the stimuli. Accordingly, we hypothesized that incongruent images would receive higher ratings than congruent images for question (a), and that the opposite pattern should be found for question (b), with congruent images rated higher than incongruent images. These hypotheses were borne out by the data in the item-based analysis, with all 120 image pairs showing significant differences between congruent and incongruent images in the expected direction for both these questions (see Fig. 5 and further details in the Supplementary Table). The group-level analyses further confirmed that the stimuli indeed met their definition as congruent and incongruent, with incongruent images rated as weirder (congruent: *M* = 1.64, *SD* = 0.36; incongruent: *M* = 4.98, *SD* = 0.46; *t*(119) = 66.63, *p* < .0001; BF_10_ > 100) and less likely to appear in the real world (congruent: *M* = 5.64, *SD* = 0.61; incongruent: *M* = 1.74, *SD* = 0.38, *t*(119) = 57.89, *p* < .0001; BF_10_ > 100) than congruent images.

*Additional rating questions*: The following questions were aimed at acquiring more data and norms on the stimuli, to be used by future researchers for different purposes, depending on their research goals. We accordingly had no hypotheses about the patterns of results, but rather looked for differences on a more exploratory basis (though these analyses were preregistered at the item-level). Between-group analyses revealed differences in ratings for questions (c) (“How visually complicated is the image?”), (d) (“How hard is it to identify the object?”), (e) (“How disturbing is the image?”), and (f) (“How much do you like the image?”). Overall, congruent images were rated as less visually complicated than incongruent images (congruent: *M* = 2.21, *SD* = 0.38; incongruent: *M* = 2.83, *SD* = 0.33, *t*(119) = 23.06, *p* < .0001; BF_10_ > 100). Subjects further reported that it was easier to identify the object for congruent images than for incongruent images (congruent: *M* = 1.50, *SD* = 0.33; incongruent: *M* = 1.74, *SD* = 0.42, *t*(119) = 6.14, *p* < .0001; BF_10_ > 100). Finally, congruent images were judged to be less disturbing (congruent: *M* = 1.5, *SD* = 0.36; incongruent: *M* = 2.31, *SD* = 0.79, *t*(119) = 10.97, *p* < .0001; BF_10_ > 100) and more liked (congruent: *M* = 3.57, *SD* = 0.61; incongruent: *M* = 2.97, *SD* = 0.49, *t*(119) = 10.95, *p* < .0001; BF_10_ > 100) than incongruent images. No difference between the conditions was found for arousal ratings, with both types of images rated as fairly non-arousing (congruent: *M* = 2.24, *SD* = 0.37; incongruent: *M* = 2.26, *SD* = 0.31; *t*(119) = 0.35, *p* = .798; BF_10_ = 0.108) (for ratings in questions (c)–(g), see again Fig. 5 and the Supplementary Table).

*Object probability question*: Question (h), “Which object is MOST likely to be found in the scene?”, was aimed at gauging subjects’ expectations about object identity given the context in which it appeared. It was not statistically analyzed, but rather used to descriptively characterize each pair in our stimulus set, again in order to allow future researchers to choose more or less constraining contexts for their studies. Following the exclusion of two subjects who reported not following the instructions for this question, we examined the answers given by all other included subjects. We found 26 additional subjects who always marked the object that appeared in the image, irrespective of its congruency with the scene (akin to the two excluded subjects). These additional subjects were accordingly removed only from the analysis of this question. For the remaining 374 subjects, in all 240 images, the congruent object was chosen as the most likely object to appear in the scene by the vast majority of subjects (*M* = 89.71%, *SD* = 6.93%; *M* here represents the mean of mean response per image). The incongruent object was not chosen as most likely to appear in any of the images. Focusing on incongruent images only, 119 images out of 120 still had the congruent object as the most common answer (*M* = 83.78%, *SD* = 10.95%), and one had the object semantically related to the congruent object as the most common answer. This metric could be used by future researchers as a means to estimate the likelihood of the object appearing in the scene, or the degree of constraint provided by the context. The distribution of answers for each image is given in the Supplementary Table.

#### Photoshop editing quality questionnaire

In this experiment, we wanted to further examine the differences between the congruent and incongruent images, focusing on the quality of the Photoshop work. Accordingly, subjects were only asked about the digital processing of the image, to avoid carryover influences from other questions. We expected subjects to nevertheless be influenced by the congruency of the image (since for incongruent scenes it is clear that digital manipulation must have been performed, as opposed to congruent ones). We accordingly cut the stimuli so that only the immediate area surrounding the critical object was seen, making it harder, in some images, to judge the meaning of the scene.

The experiment was preregistered on OSF, including the methods, sample size, and analysis plans. The preregistration can be found at: https://osf.io/htnqd/.

##### Methods

*Participants*. A total of 100 subjects took part in two versions of the questionnaire (50 subjects per version, mean age = 23.4, 28 females). Participants were students at Tel Aviv University, and all participated for course credit. All subjects first signed an online consent form. The study was approved by the ethics committee of Tel Aviv University. Sample size was determined to be equal to the main validation questionnaire, and we collected data until we reached that sample size. This number refers to valid subjects who met our exclusion criteria (see below).

*Subject exclusion*. Two additional subjects were removed from the analysis because they did not complete the questionnaire. Twenty-one more subjects were excluded from the analysis because they failed two or more of the catch questions (see Procedure section below). More specifically, out of the first 20 subjects in this questionnaire, 13 were excluded because they failed the catch questions. This initial high exclusion rate seemed to stem from the different structure of this questionnaire: while in the main validation questionnaire there were eight questions per image that appeared in random order, forcing subjects to read them, here there was only one question per image, which was always the same, except in the catch trials. Therefore, here subjects most likely assumed that the questions were always identical and simply missed the catch questions. To resolve this issue, we added a short Zoom online meeting at the beginning of the experiment that accompanied the written instructions given to subjects. In that meeting, the experimenter stressed that catch questions might appear and that subjects needed to pay attention and read the questions carefully not to miss them. This additional coaching dramatically decreased the number of subjects who failed the catch questions (out of the remaining 93 subjects who were run with the Zoom instructions, only eight failed the catch questions).

*Apparatus and stimuli*. The questionnaire was created in Qualtrics. Subjects answered the questions using their home computers. All stimuli were cut parts of the original 121 congruent and incongruent pairs of stimuli, so as to portray only the area surrounding the critical object. Accordingly, their size differed depending on the size of the object in the scene. Three additional images that were not part of the stimulus set were presented with the catch questions. In each version of the questionnaire, 121 images were presented, 60 congruent and 61 incongruent, or vice versa.

*Procedure*. The questionnaire had two versions, each presenting 121 images (for 121 pairs of images or 242 images in total). The order of the images was randomly determined within each version of the questionnaire. For each image pair, each subject saw either the congruent or the incongruent version. Each image was accompanied by one question—“How noticeable is the Photoshop work?”—which was answered using a seven-point Likert scale of 1 (*not at all*) to 7 (*very much*). Three additional images were presented with a “catch” question, which was “For quality control purposes, please select the number five here,” answered using the same scale of 1 (*not at all*) to 7 (*very much*).

##### Results

Overall, subjects’ ratings of the noticeability of the Photoshop work were not high (*M* = 2.73, *SD* = 1.94), especially when considering that subjects were informed in advance that all images were indeed digitally manipulated. Item-based analysis within each image pair revealed that this effect was found for 72 out of the 120 pairs (see details in the Supplementary Table), with five pairs receiving higher ratings for noticeable Photoshop work for the congruent version, and 67 for the incongruent version (see again Fig. 5). The exploratory between-group analyses showed that subjects tended to judge the Photoshop work as more noticeable for incongruent images (*M* = 3.70, *SD* = 0.87) than for the congruent images (*M =* 2.83, *SD =* 0.82; *t*(119) = 9.88, *p <* .0001; BF_10_ > 100). Note that in the preregistration we listed 27 of these images as potentially problematic: while we tried to minimize the influence of context so that the answers would be driven only by the quality of the editing and not the weirdness of the incongruent images, we marked these images as ones where the context could not be completely removed. When removing these image pairs, the effect decreased, though it remained highly significant (incongruent images: *M =* 3.61, *SD =* 0.88; congruent images: *M =* 2.84, *SD =* 0.82, *t*(92) = 7.63, *p <* .0001). Importantly, for 48 pairs, no differences were found in subjects’ ratings, so researchers for whom this measure is important can simply use only this subset of images.

### Additional subsets

Aside from the subset we identified, for which no low-level differences were found between congruent and incongruent images, below we suggest two additional subsets that might be useful for future researchers, as they keep additional features of the images constant.

#### Man-made versus natural objects

When characterizing our stimuli, a notable difference was that the incongruent images had more man-made objects than natural ones. Thus, we wanted to create a subset in which this potential difference was eliminated. To do so, we focused on the subset of pairs for which both congruent and incongruent objects were man-made (there was no pair for which both objects were natural). This subset includes 83 pairs. Metrics for these pairs, including the results of all analyses conducted above for the full stimulus set, can be found in the Supplementary Table.

#### Action-based incongruencies

When creating the original stimulus set, the strategy was to take the same context image, and replace the object with either the congruent object or the incongruent one. A complementary strategy would be to keep the object constant and replace the context image to create a congruent or an incongruent action for the same object. Though we did not take that approach, we were still able to identify a subset of images for which this was indeed the case. That is, we used our stimulus set to recreate pairs where the same object (though not the same exemplar) was manipulated within a congruent and an incongruent action (e.g., congruent: teacher using a marker to write on a board; incongruent: feeding a baby with a marker instead of a spoon). This subset is substantially smaller, though, consisting of only 22 pairs. Yet again, this could still be of use for researchers interested in taking this approach using our stimuli. Akin to the other subsets we created, here too we analyzed the data again for these newly created pairs. Full results can be found in the Supplementary Table.

## Discussion

In this manuscript, we introduce the ObjAct stimulus-set, which includes 120 pairs of matching congruent and incongruent images. All images portray a person performing an action with an object that can be either congruent with the action (e.g., a woman stirring a pot using a spoon) or incongruent with it (e.g., a woman stirring a pot using a paintbrush). The images are all real-life photos taken from internet sources, thus varying in multiple dimensions such as colors, complexity, setting (indoor-outdoor), and the gender of the person performing the action. The stimulus set was validated by 500 subjects (50 responses per image) in two preregistered experiments, testing the level of the incongruency of the images in each pair and providing additional ratings about different features of the images (i.e., arousal, likeability, ease of identifying the critical object, and noticeability of the digital manipulation of the images). The validation results revealed that at both the group and item levels, all incongruent images were indeed judged by subjects as weirder and less likely to be seen in the real world. Some, but not all, incongruent images were also rated as more disturbing, less liked, and more visually complicated than their congruent counterparts, with some of the objects in them rated as harder to recognize. This was also manifested by group-level differences between congruent and incongruent images in all these measures. In contrast, no group-level differences were found for arousal ratings. Finally, the additional questionnaire testing for noticeable differences in digital manipulation also yielded differences at the group level, with subjects’ ratings being higher for incongruent images than for congruent ones, even when excluding pairs where the incongruency was still evident despite the partial presentation of the cut image. This was also found at the item level for 67 of the pairs, while for five image pairs the effect was reversed. However, these ratings might still be heavily biased by semantic context, despite our efforts to neutralize it. Thus, we recommend taking the latter results with a grain of salt. Most importantly, we created a subset of 100 pairs of images in which low-level analyses revealed no difference in luminance or contrast between congruent and incongruent images, and a bottom-up, data-driven approach using DCNN mimicking the processing of the images in V1 (Krizhevsky et al., 2012) also yielded no systematic differences between congruent and incongruent images (this does not imply, of course, that no such differences exist; but we did not find them here, in either analysis type). We further identified two additional subsets that could be used by future researchers according to their needs. In the first (*N* = 83 pairs), all objects are man-made rather than natural. In the second (*N* = 22 pairs), the objects are kept constant while the context images change to create a congruent or an incongruent image. Notably, all information for the above measures—including individual ratings, low-level values, image specifications, image sources, and significance testing per item—is available at the item level in the Supplementary Table, allowing future researchers to choose images based on their ratings on the different scales, their semantic characterization, or their computed values from the low-level visual analyses.

Given the above, this stimulus set uniquely enables researchers from different labs to use well-characterized, strongly validated, and well-documented stimuli in their experiments, without having to go through the tedious process of creating and validating these stimuli. Beyond the different subsets we have identified, the elaborated classification of the images further allows great flexibility for an informed selection of relevant stimuli for each study, based on their different characteristics. In addition, the ability to access the source files from which the images were selected further enables researchers to modify the stimuli if they wish to do so (e.g., use both the modified, final images, and the isolated objects appearing in them, by using only the object layers).

We hope that the ObjAct stimulus-set will accordingly serve as a valuable resource for researchers in the community. Aside from the thorough validation processes, the ObjAct stimulus-set has two important advantages: First, human actions give rise to highly constraining contexts, as was confirmed by the results of the validation questionnaire: for all images, the congruent object was chosen by the vast majority of subjects (*M* = 89.71%, *SD* = 6.93%) as most likely to appear in the scene even when it was not presented in it. This is further corroborated by previous studies reporting better recognition and priming-related reduction in neural activation for objects following videos of hands pantomiming a congruent action compared with an incongruent one (Sim, Helbig, Graf, & Kiefer, 2015). Similarly, better recognition was found for objects that followed similarly manipulated objects (e.g., a frying pan is better recognized when appearing with a dust pan, as they are both held similarly; Helbig, Graf, & Kiefer, 2006). Interestingly, the latter effect is found only for object pictures and not object names, suggesting that visual object processing is facilitated by action-related information.

Second, the use of real-life images makes the stimuli more ecological (compared, for example, with line drawings) and, presumably, more readily processed. Indeed, previous studies found that real-life images are better learned (Evertson & Wicker, 1974), remembered (Brodeur, O’Sullivan, & Crone, 2017), and recognized (Heuer, 2016) than line drawings. The latter study also demonstrated that subjects tend to fixate more on real-life images. Similarly, manipulable objects are named faster when presented as photographs as opposed to line drawings (Brodie, Wallace, & Sharrat, 1991; Salmon, Matheson, & McMullen, 2014). Notably, this effect was not found for non-manipulable objects (see again Salmon et al., 2014), suggesting that using real-life images might be especially beneficial for processing action-related objects that are the focus of this stimulus set. Using real-life scenes seems even more crucial when studying image incongruency, since congruency is based on past experience (Bar, 2004; Biederman et al., 1982), with congruent objects being more likely to appear in the scene than incongruent objects. The more realistic the scenes, the more they mimic past experiences, arguably evoking stronger expectations.

To conclude, the ObjAct stimulus-set can serve researchers in many fields, from top-down, contextual effects on perception, through mechanisms of expectations and their violation, to action-related effects. The extensive documentation on each image, including information about low-level and high-level characteristics, will enable researchers to select specific stimuli according to their experimental needs, and to conduct highly controlled studies using this ready-made, free-to-use stimulus set.
